# Safety and Feasibility of Esophagectomy Following Combined Immunotherapy and Chemotherapy for Locally Advanced Esophageal Squamous Cell Carcinoma: A Propensity Score Matching Analysis

**DOI:** 10.3389/fimmu.2022.836338

**Published:** 2022-03-01

**Authors:** Zhi-Nuan Hong, Lei Gao, Kai Weng, Zhixin Huang, Wu Han, Mingqiang Kang

**Affiliations:** ^1^ Department of Thoracic Surgery, Fujian Medical University Union Hospital, Fuzhou, China; ^2^ Key Laboratory of Cardio-Thoracic Surgery (Fujian Medical University), Fujian Province University, Fuzhou, China; ^3^ Key Laboratory of Ministry of Education for Gastrointestinal Cancer, Fujian Medical University, Fuzhou, China; ^4^ Fujian Key Laboratory of Tumor Microbiology, Fujian Medical University, Fuzhou, China

**Keywords:** esophagectomy, neoadjuvant chemotherapy and immunotherapy, neoadjuvant chemoradiotherapy, esophageal squamous cell carcinoma, propensity score matching (PSM)

## Abstract

**Objectives:**

The combination of neoadjuvant chemotherapy and immunotherapy (nICT) is a novel treatment for locally advanced esophageal cancer. There is concern that nICT may increase operation difficulty, postoperative morbidity, and mortality. This study aimed to compare short-term outcomes among esophagectomy after neoadjuvant chemoradiotherapy (nCRT) and nICT and for locally advanced esophageal squamous cell carcinoma (ESCC).

**Methods:**

A retrospective analysis of a prospectively maintained database was performed to identify patients (from January 2017 through July 2021) who underwent surgery for ESCC following neoadjuvant therapy. A 1:1 propensity score matching (PSM) with a caliper 0.05 was conducted to balance potential bias.

**Results:**

A 1:1 PSM was conducted based on clinical stage, age, body mass index (BMI), and tumor location, and then 32 comparable pairs were matched. After PSM, age, gender, BMI, American Society of Anesthesiologists (ASA) status, smoking history, clinical stage, tumor location, lymphadenectomy field, pathological stage, anastomotic position, route of gastric conduit, procedure type, and operative approach were comparable between groups. Compared with the nICT group (median, 300 min), the operation time was significantly longer in the nCRT group (median, 376 min). However, both groups were comparable in intraoperative blood loss, thoracic drainage volume, intensive care unit (ICU) stay, postoperative hospital stays, and hospital cost. Further, 30-day mortality, 30-day readmission, ICU readmission, and major complications were similar in both groups. The nCRT group had an advantage in pathological response. The pathological complete response (pCR) was 18.8% (6/32) in the nICT group and 43.8% (14/32) in the nCRT group (*p* = 0.03). The major pathological response (MPR) was 71.9% (23/32) in the nCRT group and 34.4% (11/32) in the nICT group (*p* = 0.03).

**Conclusions:**

Based on our preliminary experience, esophagectomy appears to be safe and feasible following combined neoadjuvant immunotherapy with chemotherapy for locally advanced esophageal cancer.

## Background

Esophageal cancer (EC) is one of the most common and challenging types of cancer with 572,000 new diagnosis cases and 500,000 deaths annually. More than 50% of EC occurred in East Asia, and 90% of those patients have esophageal squamous cell carcinoma (ESCC) (1, 2). Esophagectomy plays an important role in the treatment of locally advanced EC. However, esophagectomy alone is often associated with high recurrence and metastasis rates reaching up to 43.3%–50.0% (3).

Neoadjuvant chemotherapy (nCT) or neoadjuvant chemoradiotherapy (nCRT) plus surgery had been applied to improve long-term results. Kamarajah et al. reported that nCT or nCRT followed by surgery for esophageal adenocarcinoma were equal in 5-year survival rates, and nCRT followed by surgery had an advantage in 5-year survival rates for ESCC (4). Currently, nCRT plus esophagectomy is recommended as first-line therapy for locally advanced ESCC (5). However, based on the long-term result of NEOCRTEC5010 Randomized Clinical Trial, the 5-year cumulative incidence of locoregional recurrence, distant recurrence, and overall recurrence in the nCRT group were 15.3%, 24.3%, and 32.2%, respectively, in locally advanced ESCC, which were still not promising (6). Further, a meta-analysis showed that compared with the surgery alone group, nCRT followed by surgery had an increased risk of total postoperative mortality and treatment-related mortality in patients diagnosed with ESCC (7). Thus, it is necessary to explore the novel pattern of neoadjuvant therapy for ESCC.

Based on CheckMate 577 trails results, Kelly et al. reported that nivolumab adjuvant therapy could prolong 11.4 months of disease-free survival among patients with resected esophageal or gastroesophageal junction cancer who had received nCRT (8). Further, pembrolizumab (PD-1 antagonist) plus chemotherapy has been recommended as first-line therapy for advanced EC (9). Considering the antibodies against the immune inhibitory pathway of programmed death 1 (PD-1) protein or PD-1 ligand 1 (PD-L1) checkpoint inhibitors is a new mechanism for the treatment of ESCC. nCT and immunotherapy (nICT) plus surgery may bring long-term benefits to patients diagnosed with ESCC. Recently, single-arm studies have revealed that the application of nICT in patients with locally advanced ESCC is safe and effective (10, 11).

To explore a new pattern of therapy, preoperative safety and short-term outcomes are the first concern for surgeons. However, a handful of studies focused on the comparison of short-term results and pathological response between nICT and nCRT. This study compared short-term results between nICT and nCRT to evaluate the potential effect of nICT on preoperative outcomes especially surgery safety and pathology response.

## Methods

### Patient Selection and Study Design

This is a retrospective review based on prospectively collected data conducted with consecutive patients who underwent esophagectomy followed nICT or nCRT for ESCC at Fujian Medical University Union Hospital from January 2017 to April 2021. This study was approved by the Ethics Committee of the Fujian Medical University Union Hospital, China (YF033-01). Inclusion criteria included aged between 18 and 75 years old; diagnosed with ESCC; receiving nICT or nCRT following esophagectomy; staged with cT1N1-3M0 or cT2-4aN0-3M0; and with normal hematologic, hepatic, and renal function. Exclusion criteria included patients with non-resectable tumors or metastases during exploratory surgery; patients receiving nCT or other targeted therapy; patients receiving salvage surgery; and patients with palliative surgery.

### Neoadjuvant Treatment Protocols

Patients in the nICT group received 2–4 cycles of intravenous PD-1 inhibitor (sintilimab at a dose of 200 mg, pembrolizumab at a dose of 200 mg, toripalimab at a dose of 240 mg, and camrelizumab at a dose of 200 mg) every 3 weeks (day 1) and simultaneous chemotherapy consisted of platinum-based drugs and paclitaxel (TP regimen). The details of the regimen are as follows: cisplatin (60 mg/m^2^) on day 1, albumin-bound paclitaxel (125 mg/m^2^) on days 1 and 8, and sintilimab/pembrolizumab/camrelizumab (200 mg) on day 1 were administered intravenously during each cycle (of 21-day duration). Docetaxel (75 mg/m^2^), cisplatin (60 mg/m^2^), and toripalimab (240 mg) were administered intravenously during each cycle (of 21-day duration) on day 1. Corticosteroids, thyroid function [including T3, T4, and thyroid-stimulating hormone (TSH)], glucose, and myocardial enzyme were routinely examined before each cycle of nICT for early detection of immunotherapy-related adverse events. When grade 2 above immunotherapy-related events occurred, we stopped the application of nICT and prioritized the management of adverse reactions. For patients who successfully received two cycles of neoadjuvant therapy, we conducted another clinical evaluation, followed by a multidisciplinary consultation to determine whether the patient should undergo surgery or continue treatment.

Patients in the nCRT group received chemotherapy (paclitaxel or 5-fluorouracil plus platinum) concurrent radiotherapy, which consisted of 40–56 Gy in daily fractions of 1.8 Gy five times a week. Radiation targets are mapped on CT using all diagnostic information by an experienced radiation oncologist.

### Surgery Protocols

For patients suitable for radical esophagectomy after clinical evaluation, the surgery was performed within 4–8 weeks from the end of the last neoadjuvant treatment if there were no contraindications for surgery treatment. Patients received McKeown minimally invasive esophagectomy (MIE) with or without robot-assisted, including 2-field or 3-field lymphadenectomy and gastric reconstruction. We regularly conducted 2-field lymphadenectomy, and standard three-field lymphadenectomy was performed in patients with suspected swollen lymph nodes in the neck. During the operation, patients with severe adhesion or intraoperative bleeding were transferred to thoracotomy. All operations were conducted by experienced surgeons with more than 50 cases annually, which ensured the surgery quality.

### Outcome Measures

Postoperative complications were coded by Clavien–Dindo classification, and Clavien–Dindo classification grade ≧ 3 was defined as major complications. The comprehensive complication index **(**CCI) applied as a tool to evaluate complications systematically was calculated at www.assessurgery.com (12). The primary end point was 30-day complications (including pneumonia, anastomotic leakage, pleural infusion, chylothorax, and cardiac events). Secondary end points included pathological response, operation time, postoperative thoracic drainage, thoracic drainage tube stay, 30-day readmission rate, and 30-day mortality. Interval to surgery was defined as the last measured from the end of last neoadjuvant treatment to the date of surgery, and operative time was measured from incision to wound closure. Intensive care unit (ICU) stay was defined from the day of into ICU to the day of leaving the ICU. Postoperative hospital stay was defined from the day of operation to the day of leaving Fujian Medical University Union Hospital.

The pathological TNM stage was staged according to the 8th edition of the American Joint Committee on Cancer/Union for International Cancer Control staging system. Major pathological reaction (MPR) was defined as less than 10% residual tumor cells, and pathological complete response (pCR) was defined as evidence of no residual tumor cells.

### Statistical Analysis

Patients were classified into two groups based on neoadjuvant therapy patterns. Propensity score matching (PSM) was calculated based on a logistic regression model, with a caliper 0.05, matching ratio = 1:1 to balance potential bias. The minor mirror histogram of propensity scores is listed to show the matching details (13, 14). The continuous variables were expressed as median (quartile difference), and the categorical variables were expressed as numbers (percentage). χ^2^ test was used for classification data, and Mann–Whitney U test was used for anomaly distribution data comparison. Statistical analysis was performed in R Version 4.0.4 (R Foundation for Statistical Computing, Vienna, Austria). Bilateral *p*-values <0.05 were considered significant.

## Results

### Patient Selection and Baseline Characteristics

**Figure 1:**
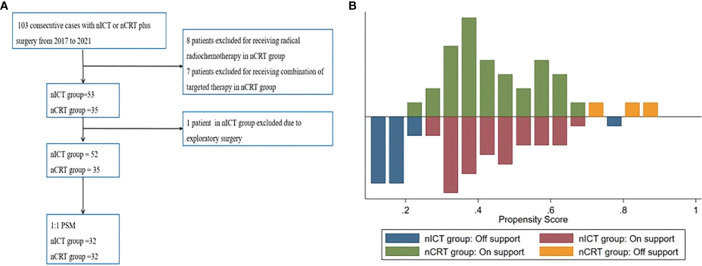
**(A)** Patient selection flowchart. **(B)** Mirror histogram of propensity scores for patients. PSM, propensity score matching; nICT, neoadjuvant chemotherapy and immunotherapy; nCRT, neoadjuvant chemoradiotherapy.

The patient selection chart and PSM details are shown in **Figure 1**. A total of 52 patients in the nICT group and 35 patients in the nCRT group were included for further analysis. Then, to balance the potential bias, a 1:1 PSM was conducted based on clinical stage, age, body mass index (BMI), and tumor location. Finally, 32 comparable pairs were matched.

The baseline characteristics after PSM with 32 patients in each group are summarized in **Table 1**. After PSM, the clinical and demographic characteristics of the two groups were well balanced, including age, gender, BMI, American Society of Anesthesiologists (ASA) status, hypertension history, smoking history, tumor location, preoperative albumin, preoperative hemoglobin, forced expiratory volume in 1 s (FEV1), ejection fractions (EF), lymphadenectomy filed, clinical stage, histologic subtype, anastomotic position, route of gastric conduit, procedure type, clinical stage, and operative approach.

**Table 1:** Baseline characteristic before and after propensity score matching.

Characteristics	Before PSM			After PSM		
	nICT	nCRT	*p*	nICT	nCRT	*p*
Number	52	35		32	32	
Age	62 (56, 67)	59 (54, 65)	0.36	62 (55, 67)	60 (54, 65)	0.68
Male	39	29	0.38	21	27	0.08
BMI	22.0 (20.3, 22.7)	22.0 (20.5, 23.7)	0.76	22.0 (19.6, 22.8)	22.0 (20.7, 23.7)	0.46
ASA			0.16			0.72
≤2	47	28		27	28	
≥3	5	7		5	4	
Diabetes	5	0	0.07	3	0	0.07
Hypertension	10	3	0.17	7	2	0.07
Smoking history	30	21	0.83	15	21	0.13
FEV1	2.6 (1.9, 3.1)	2.6 (2.4, 3.0)	0.65	2.6 (1.9, 3.1)	2.6 (2.4, 3.0)	0.76
EF%	67.7 (64.1, 71.4)	66.3 (61.9, 69.4)	0.22	68.4 (64.1, 71.5)	66.3 (61.9, 69.4)	0.19
Preoperative Hb (g/L)	125 (112, 133)	126 (113, 135)	0.82	121 (105, 133)	127 (114, 133)	0.38
Preoperative albumin (g/l)	40.2 (37.7, 43.2)	40.8 (38.1, 43.9)	0.48	39.7 (36.6, 44.0)	41.2 (39.2, 43.9)	0.18
Tumor location			0.012			0.51
Upper third	3	4		2	1	
Middle third	30	28		29	28	
Lower third	19	3		1	3	
cTNM before neoadjuvant therapy			0.12			0.43
≤2	14	15		10	13	
≥3	38	20		12	19	
Lymphadenectomy			0.07			0.27
2-field	43	23		25	21	
3-field	9	12		7	11	
Anastomotic position			1.00			1.00
Cervical	52	32		32	32	
Thoracic	0	0		0	0	
Route of gastric conduit			0.66			0.23
Posterior mediastinal	46	32		27	30	
Restro-sternal	6	3		5	2	
Procedure type			0.93			1.00
Robot-assisted	10	7		6	6	
Video-assisted	42	28		26	26	

ASA, American Society of Anesthesiologists; BMI, body mass index; nICT, neoadjuvant chemotherapy and immunotherapy; nCRT, neoadjuvant chemoradiotherapy; Hb, hemoglobin, FEV1, forced expiratory volume in 1 s; EF, ejection fractions; PSM, propensity score matching.

### Comparisons of Short-Term Outcomes

All patients in both the nICT group and nCRT group successfully received MIE without conversion into thoracotomy, although lymph nodes moved number in the nICT group (median 39) was more than that in the nCRT group (median 32). However, compared with the nICT group (319.2 ± 80.2 min), the operation time was significantly longer in the nCRT group (378.8 ± 72.7 min). Both groups were comparable in intraoperative blood loss, thoracic drainage tube stays, thoracic drainage volume, postoperative hospital stay, and hospital cost. Further, 30-day mortality, 30-day readmission, and ICU readmission were also similar in both groups. Compared with the nICT group, the nCRT group had an advantage in pathological response. The pCR was 18.8% (6/32) in the nICT group and 43.8% (14/32) in the nCRT group (*p* = 0.03). The major pathological response (MPR) was 71.9% (23/32) in the nCRT group and 34.4% (11/32) in the nICT group (*p* = 0.03). Perioperative outcomes after PSM were summarized in **Table 2**.

**Table 2:** Perioperative outcomes after propensity score matching.

Outcomes	nICT group	nCRT group	*p*
Operative time (min)	300 (270, 382)	376 (330, 413)	0.003
Converted to open surgery in thoracic procedure	0	0	NA
Intraoperative blood loss (ml)	100 (50, 100)	100 (100, 150)	0.33
Lymph nodes moved number	39 (33, 49)	32 (27, 36)	0.013
Thoracic drainage tube stays (days)	8 (7, 11)	8 (7, 10)	0.87
Thoracic drainage volume (ml)	1,235 (832, 1,625)	1,148 (550, 2,136)	0.75
ICU readmission (n)	1	0	0.31
30-day mortality (n)	0	1	0.31
30-day readmission (n)	2	4	0.39
postoperative hospital stays (days)	11 (8, 14)	9 (8, 11)	0.17
CCI	20.9 (12.2, 31.4)	22.6 (8.7, 28.9)	0.36
Hospital cost (10,000 RMB)	8.9 (8.0, 10.3)	8.2 (6.7, 10.2)	0.095
Pathological response			
pCR	6	14	0.03
pCR+MPR	11	23	0.03

ICU, intense care unit; CCI, comprehensive complication index; nICT, neoadjuvant chemotherapy and immunotherapy; nCRT, neoadjuvant chemoradiotherapy; pCR, pathological complete response; MPR, major pathological response; NA, not applicable.

**Figure 2:**
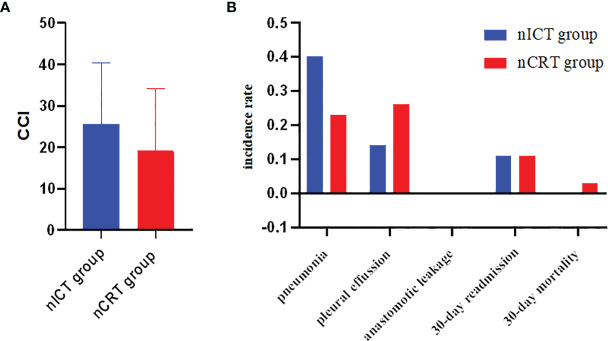
**(A)** Comparisons of CCI between nICT group and nCRT group after PSM. **(B)** Comparisons of major complications between nICT group and nCRT group after PSM. CCI, comprehensive complication index; nICT, neoadjuvant chemotherapy and immunotherapy; nCRT, neoadjuvant chemoradiotherapy; PSM, propensity score matching.

The complications within 30-day after PSM are summarized in **Table 3**. The incidence of pneumonia, pleural effusion, palsy of recurrent laryngeal nerve, chylothorax, and postoperative blood transfusion were comparable in both groups. Major complications and CCI were comparable in both groups. Comparison details of 30-day major complications and CCI after PSM are listed in **Figure 2**.

**Table 3:** Postoperative complications within 30-day after operation evaluated by Clavien–Dindo classification.

Complications	nICT Group	nCRT Group	*p*
Pneumonia			
≥Grade 2	13	9	0.29
≥Grade 3	9	7	0.33
Anastomotic leakage			
≥Grade 2	2	1	0.55
≥Grade 3	0	0	NA
Pleural effusion			
≥Grade 2	14	9	0.19
≥Grade 3	7	9	0.56
Palsy of recurrent laryngeal nerve			
≥Grade 1	0	0	NA
Cardiac events			
≥Grade 1	8	4	0.20
≥Grade 2	5	4	0.72
Chylothorax			
≥Grade 1	3	2	0.64
≥Grade 2	0	2	0.15
≥Grade 3	0	1	0.31
Postoperative blood transfusion			0.55
Grade 2	2	1	

nICT, neoadjuvant chemotherapy and immunotherapy; nCRT, neoadjuvant chemoradiotherapy; NA, not applicable.

### Immune-Related Adverse Events

**Figure 3:**
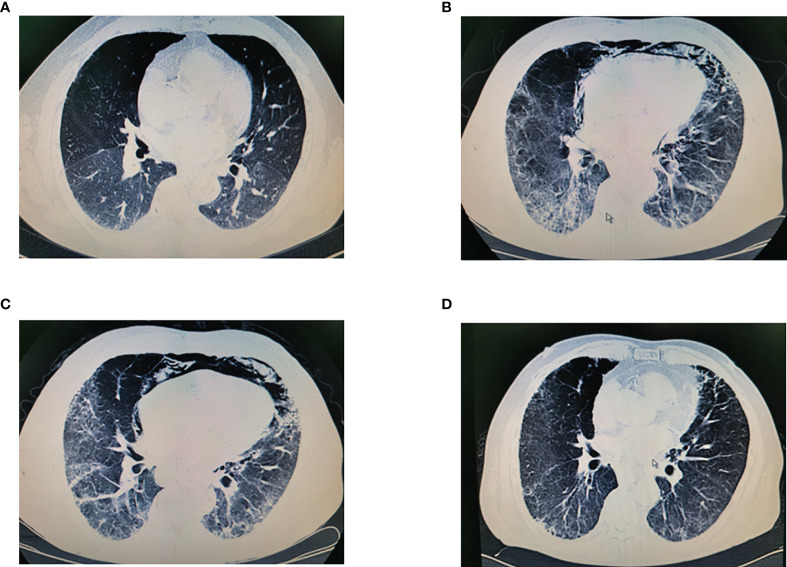
**(A)** Chest CT scan before surgery. **(B)** Chest scan on 5th day after operation. **(C)** Chest scan after application of methylprednisolone for 7 days. **(D)** Chest scan in 3 months after discharge.

Immune dermatitis was relatively common, with an incidence of 6.25% (2/32). Only one patient underwent grade 3 pneumonitis on the 10th day after the operation. This patient was admitted into ICU and treated with 40 mg of methylprednisolone for 7 days. Then, the symptoms were relieved, and this patient recovered without intubation. The chest scan changes are summarized in **Figure 3**.

## Discussion

Compared with the nICT group (median, 300 min), the operation time was significantly longer in the nCRT group (median, 376 min). The 30-day complications measured by CCI and major complications were comparable between the nICT group and nCRT group after PSM. The 30-day mortality, 30-day readmission, and ICU readmission were similar in both groups. The nCRT group had an advantage in both pCR and MPR (*p* < 0.03).

The postoperative mortality and morbidity were comparable between the nICT group and the nCRT group. Additional neoadjuvant immunotherapy did not increase postoperative mortality and morbidity, and our finding was consistent with the previous report. Sihag et al. compared the major complications between patients receiving neoadjuvant immunotherapy and chemoradiotherapy and nCRT and found that additional neoadjuvant immunotherapy was not associated with a statistically significantly increased risk of developing major complications (including pulmonary complications, anastomotic leakage, and other complications) (15). However, additional neoadjuvant immunotherapy brings additional immune-related AEs. In this study, one patient had grade 3 pneumonitis on the 7th day after the operation and finally recovered by administration of methylprednisolone treatment. A recent meta‐analysis including 12,876 patients from 23 randomized control trials showed that the incidence of pneumonitis associated with PD‐1 inhibitors was 5.17%, with an incidence of grade 3–5 pneumonitis of 4.14% (16). The time to onset pneumonitis had a really wide time window ranging from 9 days to 19.2 months (17). Pneumonia is one of the most common complications after esophagectomy. However, due to the various radiological subtypes of pneumonia, sometimes distinguishing pneumonia from pneumonitis is really a challenge. Thus, it is necessary to be vigilant for the incidence of pneumonitis when patients’ symptoms do not correspond to sputum, serum etiology, and inflammatory markers. Antibiotic treatment is recommended before pneumonitis is diagnosed.

**Figure 4:**
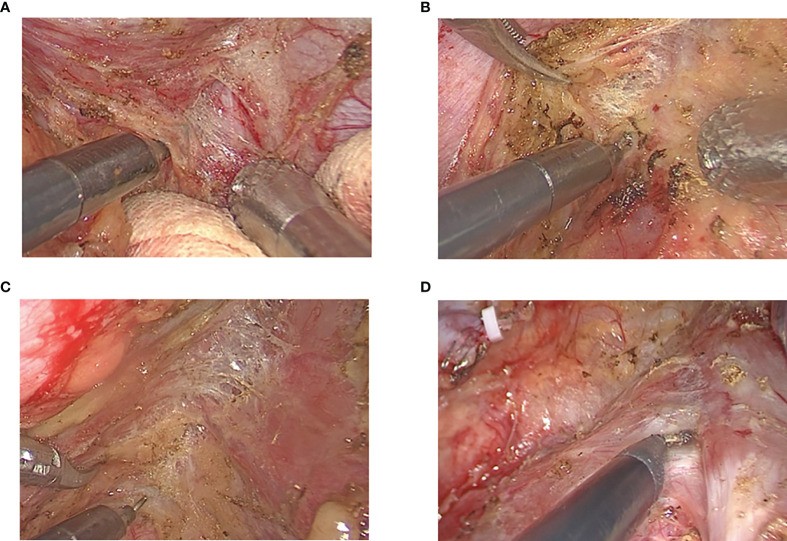
**(A)** Fibrosis in esophageal mesentery near primary tumor. **(B)** Fibrosis in esophageal mesentery near pericardium. **(C)** Fibrosis in esophageal mesentery near thoracic aorta. **(D)** Fibrosis in esophageal mesentery near trachea.

Operation time is an indicator of surgical difficulty, and moved lymph number is an indicator of surgical quality in esophagectomy. Operation time in the nCRT group was longer than that in the nCRT group, and moved lymph nodes in nICT were more than that in the nCRT group (both above 15), although previous studies indicated that surgical resection of non-small cell lung cancer (NSCLC) following neoadjuvant immunotherapy increased surgical difficulty and challenge due to dense fibrosis, especially in mediastinal and hilar dissection (18, 19). Further, there was also a possibility of unexpected conversion into thoracotomy in patients following immunotherapy (20). For patients with NSCLC, there was a reduction of moved lymph nodes in the neoadjuvant immunotherapy group (21). However, there was still no similar report in esophagectomy following nICT. In this study, all patients in the nICT group received McKeown MIE without any transformation thoracotomy. We admitted that after the application of nICT, the primary tumor shrunk, and there was fibrous scarring in the esophageal mesentery, which led to difficulty in distinguishing the boundary between the tumor and surrounding organs or tissues. Clinical practice showed that after nICT, the fibrosis was not dense, and for experienced surgeons, MIE was still applicable. For patients receiving nCRT, the primary tumor site of esophageal cancer often exhibits necrosis, fibrosis, and organizational alterations (**Figure 4**). Further, in some cases, the normal tissue gap disappears and transforms into dense fibrosis. Adhesive fibrosis is more serious in patients with obvious tumor invasion before radiotherapy (22). Thus, resection in the nCRT group is more difficult and requires more time to separate the esophagus from surrounding tissues and organs. In summary, for resection following nICT or nCRT, the surgical procedures have high technical requirements for surgeons. Such procedures should be conducted by experienced surgeons. Based on the present evidence, it seems that compared with nCRT, esophagectomy following nICT had a lower operation difficulty.

The nCRT group had an advantage in pathological response. The pCR rate was 18.8% (6/32) in the nICT group and 43.8% (14/32) in the nCRT group (*p* = 0.03). pCR was proved to be associated with long-term survival. Kamarajah et al. analyzed the National Cancer Database (2006–2015) including 2,367 with ESCC (nCRT 2,155 and nCT 212) and found that for ESCC, nCRT followed by surgery had advantage in pCR rate (50.9% versus 30.4%; *p* < 0.001), margin-negative resections (92.8% versus 82.4%, *p* < 0.001), and a statistically significant overall survival benefit (hazard ratio (HR) 0.78, 0.62 to 0.97) (23). A recent meta-analysis including 4,529 patients (nCT 2,035 and nCRT 2,494) showed that compared with nCT, nCRT could provide a higher 3-year survival benefit, higher R0 resection, and pCR rates, but no increase in 5-year survival (24). Zhang et al. conducted a propensity score-matched study from the National Cancer Center to compare the long-term results between nCRT and nCT and found that significantly higher pCR rates in the nCRT group did not lead to a longer survival (25). Thus, considering the different mechanisms of immunotherapy, it is worth conducting a long-term follow-up to evaluate the effect of nICT. Whether nICT could challenge nCRT or nCT in the first-line treatment of locally advanced ESCC requires the evaluation of long-term results in a cohort with a sufficient sample size.

To our best knowledge, this is the first study that compares the short-term outcomes between nICT and nCRT. We tried to solve the potential selection and detection bias by strict patient selection and PSM. However, this study was still limited by its retrospective nature, and it was also only conducted in a single institution with a relatively limited case number. Due to the lack of patients’ compliance and the quality control of radiotherapy, the application of nCRT was relatively limited, and nCT was a more popular pattern in China. Some patients received nCRT in other hospitals and then received surgery in our institution. Thus, the case number of the nCRT group in our institution was limited with potential bias. We used pCR to evaluate the antitumor effect of neoadjuvant therapy. Although pCR has been proved to be associated with long-term survival, long-term follow-up is necessary for further confirming the effect of nICT. To determine whether nICT could challenge the status of nCRT in the treatment of locally advanced ESCC and to establish a new treatment model based on immunotherapy, there is an urgent need for more phase III clinical trials and more convincing research results.

## Conclusion

Compared with nCRT, esophagectomy following nICT had a lower operation difficulty, similar postoperative complications, and similar mortality. Esophagectomy following nICT is safe and feasible for locally advanced ESCC. Long-term follow-up is necessary to evaluate the effect of nICT.

## Data Availability Statement

The raw data supporting the conclusions of this article will be made available by the authors, without undue reservation.

## Ethics Statement

The studies involving human participants were reviewed and approved by Fujian Medical University Union Hospital. The patients/participants provided their written informed consent to participate in this study.

## Author Contributions

Z-NH conceived the concept and coordinated the design. Z-NH drafted the manuscript with significant contributions from LG and KW. All authors listed have made a substantial, direct, and intellectual contribution to the work and approved it for publication.

## Funding

This study was sponsored by the Key Laboratory of Cardio-Thoracic Surgery (Fujian Medical University), Fujian Province University.

## Conflict of Interest

The authors declare that the research was conducted in the absence of any commercial or financial relationships that could be construed as a potential conflict of interest.

## Publisher’s Note

All claims expressed in this article are solely those of the authors and do not necessarily represent those of their affiliated organizations, or those of the publisher, the editors and the reviewers. Any product that may be evaluated in this article, or claim that may be made by its manufacturer, is not guaranteed or endorsed by the publisher.
